# Toward Sustainable Paclitaxel Bioproduction: Plant Biology, Biosynthesis and Platform Engineering

**DOI:** 10.3390/plants15111741

**Published:** 2026-06-04

**Authors:** Meng Zhang, Xing Xing, Hongliang Zhu

**Affiliations:** 1 Sichuan Advanced Agricultural & Industrial Institute, China Agricultural University, Chengdu 611430, China; zhang_meng@cau.edu.cn; 2 College of Food Science & Nutritional Engineering, China Agricultural University, Beijing 100083, China; s20243061286@cau.edu.cn

**Keywords:** paclitaxel, taxadiene synthase, cytochrome P450, baccatin III, metabolic engineering

## Abstract

Paclitaxel (Taxol), a taxane diterpenoid from Taxus species, is a clinically important microtubule-stabilizing anticancer agent widely used in chemotherapy. However, its supply remains limited by precursor scarcity and the molecule’s structural complexity. The biosynthetic pathway from geranylgeranyl diphosphate (GGPP) to paclitaxel is estimated to involve 19 to 23 enzymatic steps. Recent multi-omics approaches have substantially elucidated this pathway, yet key mechanistic questions persist, notably the formation of the oxetane ring. Complete heterologous biosynthesis is further hampered by poor cytochrome P450 (CYP) expression in non-native hosts and insufficient metabolic flux. This review synthesizes advances across four themes: (1) progressive elucidation of the biosynthetic pathway, with emphasis on the CYP-mediated oxygenation cascade and oxetane ring formation; (2) genomic and regulatory insights from *Taxus* genome assemblies, transcription factor networks, and spatial multi-omics; (3) metabolic engineering in microbial hosts, including *Escherichia coli*, *Saccharomyces cerevisiae*, and non-conventional chassis; and (4) plant-based heterologous production platforms. Critical bottlenecks are identified, including unresolved enzymatic steps, CYP functional expression, flux partitioning, and bioprocess scale-up. Strategies to overcome these challenges are discussed.

## 1. Introduction

Cancer accounted for an estimated 20 million new diagnoses and 9.7 million deaths worldwide in 2022 [1]. Among chemotherapeutic agents deployed against this burden, paclitaxel occupies a distinctive niche: it binds β-tubulin subunits, stabilizes microtubule polymers, and arrests mitosis at the G2/M phase, displaying broad efficacy against ovarian, breast, non-small-cell lung, and pancreatic carcinomas [2]. Originally isolated from the bark of the Pacific yew (*Taxus brevifolia*) in the 1960s, paclitaxel has remained among the highest-grossing oncology drugs for over three decades.

Yet the supply of paclitaxel has been fraught with difficulty from the outset. Bark stripping of wild *T. brevifolia* was ecologically destructive—approximately 10,000 kg of bark yielded merely 1 kg of product—and this supply crisis catalyzed the search for alternative production routes. Current commercial manufacturing depends mainly on two strategies: semi-synthesis from 10-deacetylbaccatin III (10-DAB III) obtained from renewable Taxus biomass and biotechnological production using Taxus cell suspension cultures [3,4,5]. The latter has become an established industrial bioproduction route because Taxus cells retain the native enzymatic, regulatory, and subcellular machinery required for taxane biosynthesis and can be cultivated under controlled bioreactor conditions [6]. Plant cell fermentation also reduces dependence on destructive bark harvesting and avoids seasonal or geographical constraints associated with field-grown Taxus biomass. Nevertheless, this platform remains limited by slow cell growth, cell-line-dependent productivity, elicitation sensitivity, long cultivation cycles, and high production costs, which continue to motivate the development of complementary heterologous and synthetic biology-based production strategies [7]. More broadly, both semi-synthesis and cell-culture-based production still face constraints in scalability, process stability, and cost efficiency. Total chemical synthesis, although accomplished through multiple elegant routes, remains commercially impractical owing to the molecule’s formidable structural complexity: a 6–8–6–4 tetracyclic (ABC–D ring) skeleton bearing eleven stereocenters, an oxetane D-ring, and an N-benzoylphenylisoserine side chain [8]. In this review, sustainable paclitaxel supply refers to production strategies that reduce pressure on natural Taxus resources, minimize ecological disturbance, improve reproducibility and scalability, and increase the economic feasibility of paclitaxel manufacturing [9]. This concept includes not only replacing destructive bark harvesting, but also improving the productivity and stability of Taxus cell cultures, developing microbial or photosynthetic chassis for taxane precursor production, and using plant-based systems to support CYP-dependent oxidative tailoring [5]. From this perspective, sustainability should be evaluated by both biological performance and process-level criteria, including pathway completeness, host compatibility, production stability, energy or resource input, downstream purification, and scalability [7,10].

These supply limitations have driven sustained efforts to reconstitute the complete paclitaxel pathway in heterologous hosts via metabolic engineering and synthetic biology. The pathway encompasses ~19 enzymatic transformations: cyclization of the universal diterpene precursor GGPP by taxadiene synthase (TXS); a multi-step oxygenation cascade catalyzed by CYP725-family cytochrome P450 monooxygenases; acyl-group transfers by BAHD-family acyltransferases; and a distinctive oxetane ring formation [2,11]. The oxygenation cascade alone involves at least eight CYP-mediated hydroxylation and epoxidation steps, each requiring electron delivery from NADPH via cytochrome P450 reductase (CPR)—a degree of CYP pathway complexity that is exceptional even among plant specialized metabolites. For much of the past two decades, only a subset of these enzymes—predominantly those catalyzing the early and late steps—had been functionally characterized, while the identities of several middle-pathway CYPs and the enzymatic logic of oxetane assembly remained unresolved. This knowledge gap constituted the principal barrier to heterologous reconstitution.

Several recent reviews have summarized paclitaxel biosynthesis, Taxus genomic resources, regulatory mechanisms, and synthetic biology-based production [3,12,13]. The present mini-review differs from these studies by focusing specifically on the acceleration of the field since 2021, when chromosome-level Taxus genomes, spatial multi-omics, structural enzymology, and combinatorial pathway reconstitution began to close long-standing gaps in the paclitaxel pathway [14,15,16,17,18]. Rather than providing a broad historical survey, we emphasize newly resolved enzymatic steps, revised pathway models, recent reconstitution studies leading to baccatin III and paclitaxel, and the remaining bottlenecks that constrain commercially viable heterologous production [7,17]. We further discuss how microbial, plant-based, photosynthetic, and Taxus cell culture platforms differ in their bottlenecks, sustainability relevance, and development priorities [10,19].

## 2. Elucidation of the Paclitaxel Biosynthetic Pathway

### 2.1. The First Committed Step: Cyclization of GGPP by Taxadiene Synthase

**Figure 1:**
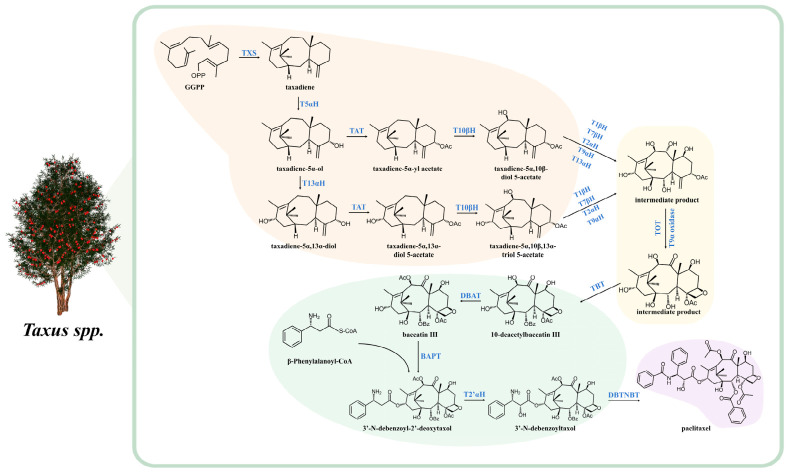
Biosynthesis pathway of paclitaxel. Paclitaxel biosynthesis begins with TXS-catalyzed cyclization of GGPP to taxadiene, followed by CYP725-mediated oxygenation, acyltransferase-catalyzed decoration, oxetane ring formation, baccatin III formation, side-chain attachment by BAPT, and N-benzoylation by DBTNBT. Enzyme labels indicate characterized or proposed catalytic steps, and arrows denote the reaction direction. A late taxane intermediate is benzoylated by TBT to form 10-deacetylbaccatin III, which is subsequently acetylated at C10 by DBAT to generate baccatin III.

Paclitaxel biosynthesis is initiated by TXS, a class II/I bifunctional diterpene cyclase that converts GGPP to taxa-4(5),11(12)-diene (taxadiene) via an ionization-dependent cyclization cascade involving intramolecular proton transfer and two sequential C–C ring closures (Figure 1). GGPP is supplied primarily by the plastid-localized methylerythritol phosphate (MEP) pathway, with potential supplementation from the cytosolic mevalonate (MVA) pathway via intercompartmental IPP/DMAPP exchange [20]. As the first committed step, TXS has been subject to extensive mechanistic investigation. Molecular dynamics simulations and mutability landscape analyses have delineated the active-site residues governing substrate orientation, product fidelity, and the inherent catalytic promiscuity of this enzyme—the latter manifesting as the formation of minor verticillene- and cembrene-type by-products [21]. These insights directly inform efforts to enhance TXS catalytic efficiency and soluble expression in heterologous hosts.

### 2.2. The CYP725-Mediated Oxygenation Cascade and Acyl-Group Decoration

From taxadiene onward, the pathway traverses an extensive oxygenation cascade catalyzed by CYP725-family P450 monooxygenases, each requiring electron delivery from a cognate NADPH–cytochrome P450 reductase (Figure 1). The first oxygenation is mediated by taxadiene 5α-hydroxylase (CYP725A4/T5αH), which catalyzes C-5 hydroxylation of taxadiene to yield taxadien-5α-ol, with concomitant generation of a minor rearrangement by-product, 5(12)-oxa-3(11)-cyclotaxane (OCT). CYP725A4 has long constituted the most recalcitrant bottleneck in heterologous reconstitution: its low catalytic turnover, propensity for misfolding in non-native membranes, and strict dependence on a compatible CPR partner collectively constrain oxygenated taxane titers [22,23]. The recent crystal structure determination of T5αH, complemented by quantum mechanics/molecular mechanics (QM/MM) modelling, has elucidated the active-site architecture responsible for its regio- and stereoselectivity, furnishing a structural template for rational redesign [24]. In a complementary approach, ancestral sequence reconstruction has been employed to generate hydroxylase variants with enhanced catalytic parameters [25], underscoring the utility of evolutionary enzyme engineering for this challenging target.

Subsequent C-13 hydroxylation is catalyzed by taxane 13α-hydroxylase, another CYP725 family member characterized as a P450-dependent monooxygenase [26]. Downstream oxygenation steps progressively introduce hydroxyl groups at the C-2, C-7, C-9, and C-10 positions of the taxane core, interspersed with acylation reactions mediated by BAHD-family acyltransferases. Three such enzymes have been biochemically characterized: DBAT (10-deacetylbaccatin III 10-*O*-acetyltransferase), which acetylates the C-10 hydroxyl to yield baccatin III; BAPT (baccatin III 3-amino-3-phenylpropanoyltransferase), which installs the β-phenylalanine-derived side chain; and DBTNBT (3′-*N*-debenzoyl-2′-deoxytaxol *N*-benzoyltransferase), which completes the *N*-benzoylation [11]. A mechanistically distinctive reaction in this pathway is the formation of the D-ring oxetane, a strained four-membered cyclic ether essential for paclitaxel’s tubulin-binding activity. A long-debated question was resolved by the demonstration that a single bifunctional CYP catalyzes both C-4β,C-20 epoxidation and the subsequent intramolecular ring closure to generate the oxetane, rather than two discrete enzymes as previously postulated [15].

### 2.3. Reconstitution of the Baccatin III Pathway and Beyond

In previous studies, roughly half of the enzymatic steps from taxadiene to paclitaxel lacked assigned enzymes, particularly those governing the middle-pathway oxygenations between taxadien-5α-ol and 10-DAB III. This impasse was broken by several convergent studies. A combination of transcriptomic co-expression analysis, heterologous enzyme reconstitution, and in vitro biochemistry identified multiple previously uncharacterized CYPs and acyltransferases, achieving the stepwise reconstitution of the pathway to baccatin III—the first heterologous production of this key tetracyclic intermediate (~50 ng/g) in *N. benthamiana* [17]. Independently, the enzymatic assembly of the highly oxygenated taxane core was elucidated through a convergent experimental strategy, corroborating and extending these findings [27].

At the early-pathway level, reconstitution of the biosynthetic network downstream of TXS uncovered unexpected pathway branching, demonstrating that the early oxygenation steps generate a broader spectrum of taxane scaffolds than the canonical linear model had predicted [28]. This finding carries direct implications for metabolic engineering: unless flux is precisely channelled toward the productive branch, off-pathway shunt products will accumulate and erode yield. The minimal gene set required for specific taxane intermediate biosynthesis was defined in *Nicotiana benthamiana*, thereby establishing a modular framework for stepwise pathway extension [29].

The final remaining gaps were closed through the identification of FoTO1, an NTF2-like scaffold protein that forms complexes with TXS and T5αOH to boost early pathway yields by 10–17-fold and additional pathway genes enabling baccatin III production at 10–30 μg/g dry weight [30], and the subsequent elucidation of the terminal enzymatic steps—including side-chain conjugation and *N*-benzoylation—which demonstrated the complete biotechnological production of paclitaxel [31]. Collectively, these achievements have transformed the paclitaxel pathway from a partially mapped route into a nearly fully defined enzymatic sequence, marking the onset of what has been described as a new era in taxane biochemistry [12,32].

## 3. Genomic and Regulatory Insights

### 3.1. Taxus Genome Assemblies and Genomics

**Figure 2:**
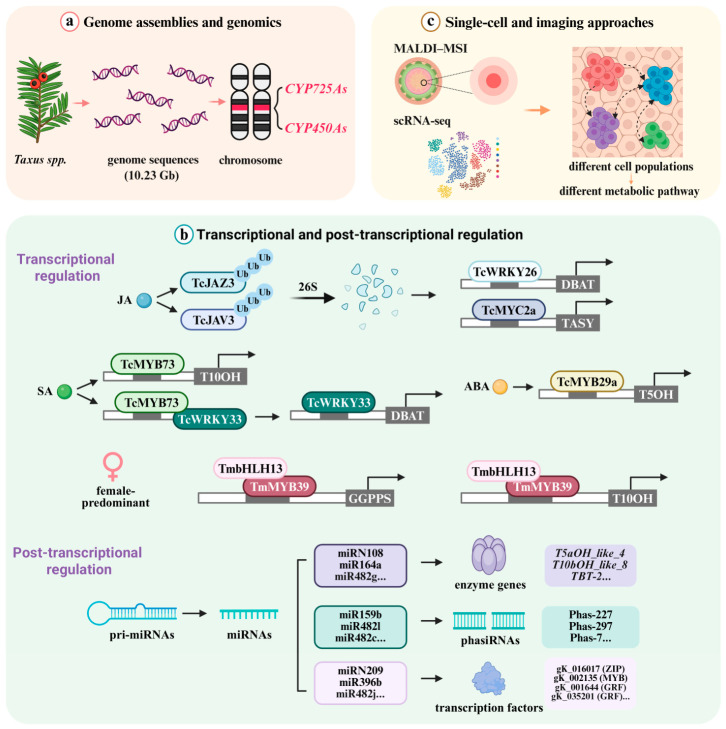
Multi-omics strategies for studying paclitaxel biosynthesis in *Taxus* spp. (**a**) Taxus genome assemblies for identifying candidate CYP725 and acyltransferase genes; (**b**) transcriptional and post-transcriptional regulatory networks involving hormone-responsive transcription factors, miRNAs, and phasiRNAs; and (**c**) single-cell transcriptomics and mass spectrometry imaging for resolving cell-type-specific taxane biosynthesis and transport.

From a plant biology perspective, paclitaxel biosynthesis represents an exceptional example of lineage-specific specialized metabolism [14,16]. Its formation requires the coordination of plastid-derived diterpene precursors, ER-localized CYP725-mediated oxidative tailoring, BAHD acyltransferase activity, hormone-responsive transcriptional regulation, and tissue- or cell-type-specific metabolic organization [33,34,35]. Gene discovery in the paclitaxel pathway has been critically enabled by the assembly of high-quality *Taxus* genomes—a technically demanding feat given the large (~10 Gb), repetitive gymnosperm genome architecture (Figure 2a). The first reference-grade *Taxus chinensis* genome revealed the clustered genomic organization of CYP725 and acyltransferase gene families and provided a systematic candidate gene catalogue for functional screening [16]. A subsequent chromosome-level assembly of the Himalayan yew (*Taxus wallichiana* var. *chinensis*) enabled reconstruction of the evolutionary trajectory of the biosynthetic pathway, demonstrating that key CYP725 paralogs arose through tandem duplication followed by neofunctionalization [14]. The *Taxus yunnanensis* genome further expanded the phylogenomic framework [36]. Collectively, these assemblies revealed that the *Taxus* CYP725 gene family has undergone extensive lineage-specific expansion, with many paralogs organized in tandem arrays on the same chromosome—a genomic signature consistent with recent, iterative duplication events that generated the enzymatic diversity required for the complex oxygenation cascade.

Comparative genomics with non-producing relatives has proven particularly illuminating. Analysis of the *Pseudotaxus chienii* genome—a closely related genus that accumulates taxane precursors but does not produce paclitaxel—pinpointed the specific gene duplication and neofunctionalization events required for the assembly of the complete biosynthetic pathway [37]. Together, these genomic resources have fundamentally expanded the functional genomic toolkit available for *Taxus* research [38] and continue to underpin the discovery of new biosynthetic and regulatory genes.

### 3.2. Transcriptional and Post-Transcriptional Regulation

Paclitaxel biosynthesis is governed by a multi-tiered regulatory network that transduces phytohormone signals into transcriptional output at biosynthetic gene promoters (Figure 2b). Jasmonate (JA) signalling serves as the principal elicitor of the pathway, consistent with its well-established role in activating plant specialized metabolism in response to biotic stress. The TcJAV3–TcWRKY26 transcriptional cascade was identified as a previously uncharacterized regulatory module linking JA perception to transcriptional activation of DBAT [39]. This cascade operates downstream of the canonical JAZ–MYC signalling module, providing an additional amplification layer specific to taxane metabolism. The discovery of this cascade illustrates the pathway-specific elaboration of JA signalling that has evolved in *Taxus* to coordinate the expression of taxane biosynthetic genes.

R2R3-MYB transcription factors function as integrative nodes that converge hormonal inputs onto biosynthetic gene promoters. TcMYB29a, an ABA-responsive activator, directly binds promoter elements of multiple pathway genes to upregulate their expression [40]. TcMYB73, responsive to salicylic acid, activates the pathway through both direct promoter binding and indirect modulation of downstream transcription factor activity, revealing a dual-mode regulatory mechanism [41]. A further stratum of specificity is conferred by the MYB39–bHLH13 complex in *Taxus media*, whose female-predominant expression underlies the sexually dimorphic accumulation of paclitaxel—an unexpected intersection between sex-biased transcription and specialized metabolism [42]. Post-transcriptionally, regulatory microRNAs and phased small interfering RNAs (phasiRNAs) fine-tune the steady-state abundance of biosynthetic gene transcripts, adding yet another layer to this regulatory architecture [43].

### 3.3. Spatial Compartmentalization Revealed by Single-Cell and Imaging Approaches

Bulk-tissue transcriptomics and metabolomics yield population-averaged data that mask the cell-type-specific organization of taxane metabolism (Figure 2c). Two recent studies have applied spatial multi-omics to resolve this heterogeneity at cellular resolution. Integration of matrix-assisted laser desorption/ionization mass spectrometry imaging (MALDI–MSI) with single-cell RNA sequencing in *Taxus mairei* stems revealed that discrete cell populations contribute to distinct pathway segments and that intercellular taxoid transport is essential for the spatial distribution of end products [34]. Analogous approaches applied to *Taxus* leaves identified tissue-specific regulatory circuits that coordinate taxane biosynthesis with leaf development [35]. Beyond fundamental insight, these spatial data expose intercellular bottlenecks—such as transport limitations and cell-type-restricted CYP expression—that must be considered when designing heterologous production strategies. Notably, the finding that biosynthetic and accumulation sites are spatially separated within *Taxus* tissues implies that successful heterologous reconstitution may require not only the core biosynthetic enzymes themselves but also the transporter proteins that mediate intercellular and subcellular taxoid trafficking.

## 4. Metabolic Engineering in Microbial Hosts

### 4.1. Escherichia coli

*Escherichia coli* served as the first microbial chassis for taxadiene biosynthesis (Table 1). The initial proof of concept was established by co-expressing TXS with a heterologous GGPP synthase (GGPPS) [44]. A pivotal subsequent advance was the introduction of a synthetic MVA pathway into *E. coli*, which dramatically enhanced IPP/DMAPP availability by decoupling precursor supply from native MEP regulation [45]. Compared with relying solely on the native MEP pathway of E. coli, introducing a heterologous MVA pathway provides a more modular and tunable route for increasing isoprenoid precursor supply. This strategy increases the availability of IPP and DMAPP, thereby supporting GGPP formation, the direct precursor of taxadiene [45,46]. Taxadiene titers have since been progressively improved through iterative optimization of promoter strength, ribosome binding site architecture, codon harmonization, and fed-batch fermentation [47,48]. The construction of a TXS–GGPPS fusion protein was shown to enhance substrate channelling and reduce GGPP pool diversion, thereby mitigating the spatial disconnect between sequential catalytic steps [48].

Extending the pathway beyond taxadiene in *E. coli* requires functional expression of eukaryotic CYP enzymes—a challenge compounded by the absence of an endoplasmic reticulum in this host. The *Taxus* CYP725 oxygenases are type II microsomal P450s that depend on ER membrane insertion for proper folding and catalytic competence, rendering their expression in a prokaryote inherently problematic. This barrier was partially overcome through systematic optimization of CYP725A4–CPR protein stoichiometry, N-terminal membrane-anchor truncation, and co-translational folding, achieving a fivefold increase in oxygenated taxane titers to ~570 ± 45 mg/L [22]. Subsequent integration of enzyme engineering with metabolic balancing reconstructed the early-stage pathway, producing multiple early oxygenated intermediates [19]. Notwithstanding these advances, the intrinsic absence of endomembrane compartments in *E. coli* fundamentally constrains the functional co-expression of the numerous ER-associated CYPs required for the downstream oxygenation cascade, positioning this host as best suited for early-pathway intermediate production rather than full pathway reconstitution.

### 4.2. Saccharomyces cerevisiae

*Saccharomyces cerevisiae* offers three compelling advantages over *E. coli* for paclitaxel pathway assembly: an endoplasmic reticulum for microsomal CYP folding, a native MVA pathway, and a mature genetic toolkit for multi-gene pathway engineering. Early work demonstrated the expression of multiple taxol pathway genes in yeast [60] and established the initial taxadiene-producing strains with a titer of ~8.7 mg/L [49]. Taxadiene titers were subsequently raised to 129 ± 15 mg/L through multi-copy chromosomal integration of TXS fused with solubility tags, combined with MVA pathway upregulation and low-temperature (20 °C) cultivation (Table 1) [50].

A central focus has been alleviating the CYP725A4 bottleneck. CPR partner compatibility, lipid membrane composition, and haem cofactor availability have been evaluated as determinants of CYP725A4 activity in yeast [23]. Oxygenated and acetylated taxane precursor titers were improved through two-phase fermentation with dodecane overlay, enhancing product partitioning and achieving up to ~78 mg/L oxygenated taxanes [51]. Genome-scale metabolic modelling coupled with combinatorial pathway engineering identified flux-limiting nodes and achieved a taxadiene titer of ~215 mg/L [52], while subsequent work achieved further taxadiene titer gains [61]. Additional strategies have targeted TXS expression via promoter and copy-number optimization [62] and elimination of enzyme catalysis compartmentalization via a GGPPS-TXS fusion enzyme, which increased the taxadiene titer by 54% and ultimately reached 184.2 mg/L in fed-batch bioreactor cultivation [53]. Despite this considerable progress, heterologous production of baccatin III or more advanced intermediates in yeast has not yet been demonstrated, owing principally to the difficulty of functionally co-expressing the full suite of *Taxus* CYPs with matched CPR partners and achieving coordinated electron transfer.

### 4.3. Non-Conventional Microbial Chassis

Several alternative microbial hosts address specific limitations of *E. coli* and *S. cerevisiae*. *Bacillus subtilis*—a Gram-positive, GRAS-status organism with robust protein secretion capacity—has been engineered for taxadiene production, achieving a titer of 17.8 mg/L (Table 1) [54]. The oleaginous yeast Yarrowia lipolytica, endowed with a high native flux through acetyl-CoA and tolerance to hydrophobic terpenoids, has also attracted attention as an alternative chassis. A taxadiene titer of 101.4 mg/L was achieved in this host by improving TXS solubility via fusion solubilizing tags and iterative multi-copy chromosomal integration [55], while peroxisome-targeted pathway engineering in this host established strategies potentially transferable to taxane biosynthesis [63].

Photoautotrophic chassis offer the distinctive advantage of coupling terpenoid biosynthesis directly to photosynthetic carbon fixation. Photosynthetic electron transport in the cyanobacterium *Synechocystis* sp. PCC 6803 was shown to power CYP-catalyzed oxygenation reactions, bypassing the need for exogenous NADPH regeneration systems [64]. This concept was extended by engineering cyanobacterial strains that produce taxadien-5α-ol at 4.32 mg/L directly from CO_2_, thereby integrating natural product biosynthesis with carbon capture [56]. In a distinct approach, taxadiene production was demonstrated in the Taxus-associated endophytic fungus Alternaria alternata TPF6 by engineering the mevalonate pathway and introducing taxadiene synthase, yielding 61.9 ± 6.3 μg/L taxadiene in the engineered strain GB127. This study established an endophytic fungal chassis for early taxane precursor production [47].

## 5. Plant-Based Heterologous Production

### 5.1. Model Plant Chassis for Taxane Pathway Reconstitution

Plant expression systems provide a cellular environment intrinsically compatible with plant-derived biosynthetic machinery, including plastidial and ER compartments, appropriate membrane lipid compositions, and native CYP–CPR redox coupling. Taxadiene accumulation was first demonstrated in transgenic *Arabidopsis thaliana* by redirecting plastidial GGPP flux toward TXS, achieving ~600 ng/g dry weight (Table 1) [57]. Taxadiene production was subsequently achieved in the moss *Physcomitrella patens*, establishing an alternative genetically tractable platform [65].

The most substantial advances have exploited *Nicotiana* species as chassis. The amenability of *N. benthamiana* to *Agrobacterium*-mediated transient expression allows rapid combinatorial testing of multi-gene pathway modules without stable transformation, substantially accelerating the design–build–test cycle. Committed taxane biosynthesis was demonstrated in *N. benthamiana* by co-engineering the chloroplastic MEP pathway to enhance GGPP supply while simultaneously expressing downstream taxane-modifying enzymes [33]. Chloroplast-targeted TXS expression markedly increased taxadiene accumulation to 56.6 ± 3.2 μg/g fresh weight in tobacco, consistent with the hypothesis that co-localizing TXS with the MEP-derived GGPP pool minimizes substrate competition with endogenous diterpene pathways [58]. A modular synthetic biology strategy was then adopted, systematically co-expressing defined gene combinations in *N. benthamiana* to identify the minimal gene set sufficient for the production of key taxane intermediates [29]. Recent studies have identified multiple missing pathway genes and achieved baccatin III biosynthesis in *N. benthamiana* [17,30]. These advances establish plant chassis as realistic platforms for producing advanced taxane intermediates and may ultimately support paclitaxel biosynthesis itself [31].

### 5.2. Taxus Cell Culture Optimization and in Planta Engineering

*Taxus* cell suspension cultures represent the most mature plant-based bioproduction platform for paclitaxel because they retain the complete native biosynthetic machinery, including pathway enzymes, regulatory circuits, and intracellular compartmentation. Unlike heterologous systems, *Taxus* cells do not require complete reconstruction of the pathway from individual genes, and their productivity can instead be improved through cell-line selection, elicitation, precursor feeding, culture optimization, and targeted metabolic or regulatory engineering [5]. Complementing heterologous approaches, targeted engineering of native *Taxus* cell cultures—which natively harbour the complete biosynthetic machinery—offers a more immediate route to improved paclitaxel titers (Table 1). Overexpression of BAPT and DBTNBT in Taxus baccata in vitro cultures enhanced paclitaxel accumulation under dual elicitation with coronatine and randomly methylated-β-cyclodextrins. The DBTNBT-overexpressing line produced 310 mg/L paclitaxel compared with 71.01 mg/L in the wild-type line, while the BAPT-overexpressing line produced 135 mg/L [59]. An orthogonal genome-level strategy generated *T. baccata* tetraploid lines via colchicine-induced polyploidy [66]. These native-host strategies circumvent the challenges of heterologous CYP expression and are deployable with existing cell culture infrastructure [3]. Critically, the regulatory insights discussed furnish actionable engineering targets: overexpression of JA-responsive activators such as TcMYB29a, or manipulation of the TcJAV3–TcWRKY26 cascade, could synergize with gene overexpression approaches to further elevate yields in *Taxus* cell lines.

## 6. Current Challenges and Future Perspectives

Despite the extraordinary progress documented above, three principal challenges must be addressed before heterologous paclitaxel production can be realized at commercial scale. Because these challenges differ substantially among production platforms, a platform-level comparison is necessary to clarify which systems are most suitable for near-term production, pathway discovery, or long-term sustainable biomanufacturing. As summarized in Table 2, *Taxus* cell suspension cultures remain the most mature near-term platform because they retain the native taxane biosynthetic machinery and are compatible with bioreactor-based production, although their performance is still limited by slow growth, variable productivity, and high production costs [5]. Microbial hosts such as *Escherichia coli* and *Saccharomyces cerevisiae* offer rapid engineering cycles and fermentation scalability, but their use is currently strongest for early taxane intermediates because complete pathway reconstruction is constrained by ER-type CYP expression, CPR compatibility, electron transfer, pathway branching, and taxane toxicity [22,50]. Plant-based systems, particularly *Nicotiana benthamiana*, provide a more compatible cellular environment for CYP-dependent oxidative tailoring and are powerful for pathway validation, whereas photosynthetic chassis represent a longer-term concept for CO_2_-linked production [17,18,56]. This comparison indicates that sustainable paclitaxel bioproduction will probably require a staged or hybrid strategy rather than reliance on a single universal chassis [5,7]. First and foremost, functional heterologous expression of the CYP725-family oxygenases remains the central technical bottleneck. These ER-localized enzymes exhibit low catalytic turnover, poor folding efficiency outside their native membrane environment, and stringent requirements for cognate CPR electron donors [22,23]. Promising mitigation strategies include rational active-site redesign guided by crystal structures [24], ancestral sequence reconstruction to access thermostable variants [21,25], optimization of CYP:CPR stoichiometry through ribosome binding site tuning, and lipid membrane engineering to improve CYP insertion and folding. Nevertheless, the coordinated functional expression of multiple CYPs within a single host cell—each requiring matched electron transfer—remains a formidable unsolved problem.

Second, pathway flux partitioning presents an equally critical challenge. The paclitaxel biosynthetic network is not a simple linear sequence but a branched metabolic grid that generates numerous taxane shunt products at multiple pathway nodes [20,28]. Channelling flux selectively through the productive branch while suppressing off-pathway diversions demands sophisticated control mechanisms, including dynamic gene expression circuits (for example, biosensor-actuated feedback loops), spatial enzyme co-localization via synthetic protein scaffolds or organelle targeting, and computational flux balance analysis to identify and relieve stoichiometric constraints.

Third, the transition from laboratory demonstration to industrial bioprocess introduces challenges in strain genetic stability, fermentation scalability, product toxicity management, and downstream purification. In microbial hosts, the intracellular accumulation of hydrophobic taxane intermediates can compromise membrane integrity and exert cytotoxic effects, necessitating countermeasures such as in situ product removal (ISPR), two-phase extractive fermentation, or engineering of efflux transporters. In plant cell culture systems, maintaining consistent productivity across prolonged cultivation cycles and in scaled-up bioreactors remains challenging. These bioprocess engineering dimensions have received comparatively less attention but will be indispensable for commercial translation.

Several converging technological developments are poised to accelerate progress. The comprehensive collection of *Taxus* genomes now available [14,16,36], including comparative analyses with non-producing relatives [37], provides a complete biosynthetic parts list. Single-cell transcriptomics and spatial metabolomics [34,35] continue to uncover cell-type-specific regulatory logic and candidate transport genes. Machine learning-guided enzyme design, high-throughput combinatorial pathway assembly, and cell-free biosynthesis platforms offer powerful tools for compressing the design–build–test–learn cycle. Looking further ahead, hybrid production strategies—coupling the high isoprenoid precursor flux achievable in microbial hosts with the enzymatic compatibility of plant expression systems for complex CYP-dependent transformations—may represent the most pragmatic route to commercially viable paclitaxel biosynthesis.

## 7. Conclusions

Recent years of research have reshaped the landscape of paclitaxel biosynthesis studies. The near-complete elucidation of the enzymatic pathway—underpinned by chromosome-level *Taxus* genomes and propelled by advances in functional genomics, structural enzymology, and synthetic biology—has brought the long-sought goal of heterologous paclitaxel production within realistic reach. Metabolic engineering campaigns across diverse host platforms, from *E. coli* and *S. cerevisiae* to *N. benthamiana* and cyanobacteria, have achieved progressively more complex pathway reconstitutions, while multi-layered regulatory studies have illuminated how taxane flux is controlled. Critical challenges persist—most notably in CYP functional expression, pathway flux partitioning, and bioprocess scale-up—but the rapid pace of discovery and the growing power of synthetic biology tools provide a compelling basis for optimism. A diversified production strategy, leveraging microbial hosts for high-flux precursor supply, plant platforms for CYP-dependent tailoring reactions, and engineered *Taxus* cell cultures for near-term yield gains, is likely the most effective path forward. Beyond resolving the supply problem, the successful heterologous production of paclitaxel would enable the biosynthesis of novel taxane analogs with tailored pharmacological profiles, potentially broadening the therapeutic impact of this extraordinary class of plant specialized metabolites. As the convergence of genomics, enzymology, and synthetic biology continues to accelerate, the prospect of a sustainable, scalable, and economically viable bioproduction of paclitaxel appears within reach.

## Figures and Tables

**Table 1:** Taxane biosynthesis in engineered chassis.

Chassis		Product	Yield	Approach	References
Microbial	*Escherichia coli*	Oxygenated taxanes	570 ± 45 mg/L	Systematic optimization of CYP725A4–CPR protein stoichiometry, N-terminal membrane-anchor truncation, and co-translational folding	[22]
	*Alternaria alternata*	Taxadiene	61.9 ± 6.3 μg/L	Co-overexpression of *IDI*, *tHMGR*, and *TXS* in *Taxus*-associated endophytic fungus	[47]
	*Saccharomyces cerevisiae*	Taxadiene	8.7 mg/L	Co-expression of *tHMG*, mutant regulatory protein *UPC2-1*, *GGPPS*, and codon-optimized *T. chinensis* TXS	[49]
		Taxadiene	129 ± 15 mg/L	Multi-copy chromosomal integration of TXS fused with solubility tags, combined with MVA pathway upregulation and low-temperature (20 °C) cultivation	[50]
		Oxygenated and acetylated taxanes	~78 mg/L	Two-phase fermentation with dodecane overlay to enhance product partitioning	[51]
		Taxadiene	~215 mg/L	Genome-scale metabolic modelling coupled with combinatorial pathway engineering to identify flux-limiting nodes	[52]
		Taxadiene	184.2 mg/L	Elimination of enzyme catalysis compartmentalization via a GGPPS–TXS fusion enzyme in fed-batch bioreactor cultivation	[53]
	*Bacillus subtilis*	Taxadiene	17.8 mg/L	Overexpression of MEP pathway genes, *GGPPS*, and *TXS*	[54]
	*Yarrowia lipolytica*	Taxadiene	101.4 mg/L	Improving TXS solubility via fusion solubilizing tags and iterative multi-copy chromosomal integration	[55]
	*Synechocystis*	Taxadien-5α-ol	4.32 mg/L	Co-expression of heterologous *GGPPS* and *TXS* in a high-density cultivation system with membrane-mediated CO_2_ supply	[56]
Plant-Based	*Nicotiana benthamiana*	Baccatin III	~50 ng/g	Transient expression of *TOT*, *T9αH*, and seven other known pathway genes (*TXS*, *T5αH*, *T13αH*, *T2αH*, *T7βH*, *TAT*, *TBT*)	[17]
	*Arabidopsis thaliana*	Taxadiene	~600 ng/g	Transgene expression using a glucocorticoid-mediated system; redirecting plastidial GGPP flux toward TXS	[57]
	*Nicotiana tabacum*	Taxadiene	56.6 ± 3.2 μg/g	Chloroplast-targeted TXS expression to co-localize with the MEP-derived GGPP pool, minimizing substrate competition	[58]
	*Taxus baccata*	Paclitaxel	~4-fold increase	Overexpression of late-pathway acyltransferase genes *BAPT* and *DBTNBT* in vitro cultures	[59]

*TXS*: taxadiene synthase; *T5αH*: taxadiene 5α-hydroxylase; *TAT*: taxadien-5α-ol *O*-acetyltransferase; *DBAT*: 10-deacetylbaccatin III-10-*O*-acetyltransferase; *BAPT*: baccatin III 3-amino, 3-phenylpropanoyltransferase; *DBTNBT*: 3′-*N*-debenzoyl-2-deoxytaxol *N*-benzoyltransferase; *GGPPS*: geranylgeranyl diphosphate synthase; *IDI*: isopentenyl diphosphate isomerase; *DXS*: 1-deoxy-d-xylulose-5-phosphate synthase; *DXR*: 1-deoxy-d-xylulose-5-phosphate reductoisomerase; *HMGR*: 3-hydroxy-3-methylglutaryl-CoA reductase; ERG20: farnesyl diphosphate synthase; *ERG9*: squalene synthase.

**Table 2:** Comparison of paclitaxel production platforms, bottlenecks and sustainability relevance.

Production Platform	Representative Progress	Main Contribution	Key Limitation	Sustainability Relevance	Development Priority	References
*Taxus* cell suspension culture	Taxus cell suspension cultures provide an industrially relevant plant-cell platform retaining the native taxane biosynthetic pathway.	Most mature near-term plant-based production route; does not require full heterologous pathway reconstruction.	Slow growth, variable productivity, elicitation dependence, high cost, and long-term culture instability.	Reduces destructive bark harvesting and supports controlled bioreactor-based production.	Near-term improvement of current industrial bioproduction.	[4,5]
*Escherichia coli*	Modular isoprenoid pathway optimization enabled high-level taxadiene production; P450-mediated Taxol precursor synthesis has also been optimized.	Fast growth, low-cost fermentation, and highly developed metabolic engineering tools.	Poor compatibility with ER-localized plant CYPs; lacks native endomembrane structures for complex oxidative tailoring.	Useful for scalable production of early taxane intermediates rather than complete paclitaxel biosynthesis.	Early-pathway precursor and taxadiene production.	[22,46]
*Saccharomyces cerevisiae*	Engineered yeast strains produced taxadiene at 129 ± 15 mg/L; oxygenated and acetylated Taxol precursors have been improved by advanced bioprocessing.	Eukaryotic host with ER membranes and native MVA pathway; more compatible with plant CYP expression than bacteria.	Coordinated expression of multiple CYP725 enzymes, CPR matching, and electron transfer remain difficult.	Provides a scalable fermentation platform for oxygenated taxane intermediates.	Mid-term extension from taxadiene to oxygenated intermediates.	[50,51]
*Yarrowia lipolytica*	Taxadiene production reached 101.4 mg/L after improving TXS solubility, copy number, and fed-batch fermentation conditions.	Oleaginous yeast with high acetyl-CoA flux, lipid-rich intracellular environment, and tolerance to hydrophobic products.	Taxane pathway reconstruction is less developed than in *S. cerevisiae*; downstream CYP modules remain underexplored.	Potential chassis for hydrophobic diterpenoid and taxane intermediate production.	Platform development for taxane and terpenoid biosynthesis.	[55,67]
Cyanobacteria	Engineered Synechocystis produced Taxol precursors directly from CO_2_.	Photosynthetic chassis linking taxane precursor biosynthesis with carbon fixation.	Low titers, slow growth, and limited capacity for complex multi-step taxane pathway assembly.	Long-term potential for CO_2_-based and light-driven biomanufacturing.	Long-term sustainable route for early oxygenated taxanes.	[56]
*Nicotiana benthamiana* transient expression	Chloroplastic engineering supported committed taxane biosynthesis; heterologous reconstitution enabled production of baccatin III or advanced taxane intermediates.	Plant cellular context supports plastid/ER compartmentation and CYP-dependent oxidative tailoring.	Transient expression scale-up, product recovery, and yield stability remain challenging.	Powerful platform for enzyme discovery, pathway reconstruction, and plant-compatible CYP testing.	Pathway validation and modular reconstruction.	[17,31]
Stable plant chassis	Transgenic Arabidopsis thaliana expressing taxadiene synthase demonstrated taxadiene production by redirecting plastidial GGPP flux.	Renewable biomass-based production with native plant compartmentalization.	Long transformation cycle, regulatory complexity, product recovery issues, and potential growth penalties.	Long-term renewable plant biomanufacturing concept.	Long-term engineering rather than near-term industrial production.	[57]

## Data Availability

No new data were created for the production of this manuscript. All of the data discussed and presented here are available in the relevant references cited and listed.

## References

[1] Bray F., Laversanne M., Sung H., Ferlay J., Siegel R.L., Soerjomataram I., Jemal A. (2024). Global cancer statistics 2022: GLOBOCAN estimates of incidence and mortality worldwide for 36 cancers in 185 countries. CA Cancer J. Clin..

[2] Croteau R., Ketchum R.E., Long R.M., Kaspera R., Wildung M.R. (2006). Taxol biosynthesis and molecular genetics. Phytochem. Rev..

[3] Coombe-Tennant T., Zhu X., Wu S., Loake G.J. (2025). Recent advances in paclitaxel biosynthesis and regulation. J. Exp. Bot..

[4] Tabata H. (2004). Paclitaxel production by plant-cell-culture technology. Adv. Biochem. Eng. Biotechnol..

[5] Yin J.Y., Lai M., Yu X.Y., Su D.D., Xiong X.Y., Li Y.L. (2025). Comprehensive strategies for paclitaxel production: Insights from plant cell culture, endophytic microorganisms, and synthetic biology. Hortic. Res..

[6] Xu J., Ge X., Dolan M.C. (2011). Towards high-yield production of pharmaceutical proteins with plant cell suspension cultures. Biotechnol. Adv..

[7] Tan C.L., Yu X., Feng H.C., Gershenzon J., Liu Y., Li S.H. (2026). A synthetic biology roadmap for sustainable production of the plant-originated anti-cancer drug paclitaxel. Trends Biotechnol..

[8] Min L., Han J.C., Zhang W., Gu C.C., Zou Y.P., Li C.C. (2023). Strategies and lessons learned from total synthesis of Taxol. Chem. Rev..

[9] Zerbe P. (2024). Plants against cancer: Towards green Taxol production through pathway discovery and metabolic engineering. aBIOTECH.

[10] Xie X., Zhai Y., Cheng H., Wei W.H., Ren M. (2025). From *Taxus* to paclitaxel: Opportunities and challenges for urban agriculture to promote human health. Plant Physiol. Biochem..

[11] Wang T., Li L., Zhuang W., Zhang F., Shu X., Wang N., Wang Z. (2021). Recent research progress in Taxol biosynthetic pathway and acylation reactions mediated by *Taxus* acyltransferases. Molecules.

[12] Fernie A.R., Liu F., Zhang Y. (2024). Post-genomic illumination of paclitaxel biosynthesis. Nat. Plants.

[13] Chen Q., Xin Q., Dong S., Ge X., Men X., Zhang H. (2025). Recent advances and perspectives in biosynthesis of paclitaxel: Key enzymes and intermediates. Int. J. Biol. Macromol..

[14] Cheng J., Wang X., Liu X., Zhu X., Li Z., Chu H., Wang Q., Ma Y. (2021). Chromosome-level genome of Himalayan yew provides insights into the origin and evolution of the paclitaxel biosynthetic pathway. Mol. Plant.

[15] Zhao Y., Liang F., Xie Y., Duan Y.T., Andreadelli A., Pateraki I., Makris A.M., Pomorski T.G., Staerk D., Kampranis S.C. (2024). Oxetane ring formation in Taxol biosynthesis is catalyzed by a bifunctional cytochrome P450 enzyme. J. Am. Chem. Soc..

[16] Xiong X., Gou J., Liao Q., Li Y., Zhou Q., Bi G., Li C., Du R., Wang X., Sun T. (2021). The *Taxus* genome provides insights into paclitaxel biosynthesis. Nat. Plants.

[17] Jiang B., Gao L., Wang H., Sun Y., Zhang X., Ke H., Liu S., Ma P., Liao Q., Wang Y. (2024). Characterization and heterologous reconstitution of *Taxus* biosynthetic enzymes leading to baccatin III. Science.

[18] Shi J., Zhang C., Deng R., Fernie A.R., Chen M., Zhang Y. (2025). The biosynthesis and diversity of taxanes: From pathway elucidation to engineering and synthetic biology. Plant Commun..

[19] Xie W.L., Sun C.Y., Li C.X., Zheng G.W., Xu J.H. (2026). Reconstruction of the early stage paclitaxel biosynthesis pathway in *Escherichia coli* by integrating enzyme and metabolic engineering approaches. ACS Sustain. Chem. Eng..

[20] Gou Y., Jiang X., Lian J. (2024). Intricate metabolic network for paclitaxel biosynthesis. BioDesign Res..

[21] He S., Abdallah I.I., van Merkerk R., Quax W.J. (2024). Insights into taxadiene synthase catalysis and promiscuity facilitated by mutability landscape and molecular dynamics. Planta.

[22] Biggs B.W., Lim C.G., Sagliani K., Shankar S., Stephanopoulos G., De Mey M., Ajikumar P.K. (2016). Overcoming heterologous protein interdependency to optimize P450-mediated Taxol precursor synthesis in *Escherichia coli*. Proc. Natl. Acad. Sci. USA.

[23] Nowrouzi B., Lungang L., Rios-Solis L. (2022). Exploring optimal Taxol CYP725A4 activity in *Saccharomyces cerevisiae*. Microb. Cell Fact..

[24] Song X., Wang Q., Zhu X., Fang W., Liu X., Shi C., Chang Z., Jiang H., Wang B. (2024). Unraveling the catalytic mechanism of taxadiene-5α-hydroxylase from crystallography and computational analyses. ACS Catal..

[25] Zhang Y., Wiese L., Fang H., Alseekh S., Perez de Souza L., Scossa F., Molloy J.J., Christmann M., Fernie A.R. (2023). Synthetic biology identifies the minimal gene set required for paclitaxel biosynthesis in a plant chassis. Mol. Plant.

[26] Jennewein S., Rithner C.D., Williams R.M., Croteau R.B. (2001). Taxol biosynthesis: Taxane 13α-hydroxylase is a cytochrome P450-dependent monooxygenase. Proc. Natl. Acad. Sci. USA.

[27] Yang C., Wang Y., Su Z., Xiong L., Wang P., Lei W., Yan X., Ma D., Zhao G., Zhou Z. (2024). Biosynthesis of the highly oxygenated tetracyclic core skeleton of Taxol. Nat. Commun..

[28] Liu J.C.T., De La Peña R., Tocol C., Sattely E.S. (2024). Reconstitution of early paclitaxel biosynthetic network. Nat. Commun..

[29] Zhang C., Chen W., Dong T. (2023). Elimination of enzyme catalysis compartmentalization enhancing taxadiene production in *Saccharomyces cerevisiae*. Front. Bioeng. Biotechnol..

[30] McClune C.J., Liu J.C.-T., Wick C., De La Peña R., Lange B.M., Fordyce P.M., Sattely E.S. (2025). Discovery of FoTO1 and Taxol genes enables biosynthesis of baccatin III. Nature.

[31] Liang F., Xie Y., Zhang C., Zhao Y., Motawia M.S., Kampranis S.C. (2025). Elucidation of the final steps in Taxol biosynthesis and its biotechnological production. Nat. Synth..

[32] Liu X., Zhu X., Cheng J., Jiang H. (2024). A new era for paclitaxel biosynthesis is coming. Mol. Plant.

[33] Li J., Mutanda I., Wang K., Yang L., Wang J., Wang Y. (2019). Chloroplastic metabolic engineering coupled with isoprenoid pool enhancement for committed taxanes biosynthesis in *Nicotiana benthamiana*. Nat. Commun..

[34] Yu C., Hou K., Zhang H., Liang X., Chen C., Wang Z., Wu Q., Chen G., He J., Bai E. (2023). Integrated mass spectrometry imaging and single-cell transcriptome atlas strategies provide novel insights into taxoid biosynthesis and transport in *Taxus mairei* stems. Plant J..

[35] Zhan X., Qiu T., Zhang H. (2023). Mass spectrometry imaging and single-cell transcriptional profiling reveal the tissue-specific regulation of bioactive ingredient biosynthesis in *Taxus* leaves. Plant Commun..

[36] Song C., Fu F., Yang L. (2021). *Taxus yunnanensis* genome offers insights into gymnosperm phylogeny and taxol production. Commun. Biol..

[37] Wang M., Ma R., Fang Z., Zhang L., Zhang Y., Zheng M., Bai E., Lin W., Pei Y., Zang Y. (2026). Analysis genome of *Pseudotaxus chienii* reveals insights into the origin and evolution of taxane biosynthesis. Nat. Commun..

[38] Kui L., Majeed A., Dong Y. (2022). Reference-grade *Taxus* genome unleashes its pharmacological potential. Trends Plant Sci..

[39] Chen L., Wu L., Yang L., Yu H., Huang P., Wang Y. (2022). TcJAV3-TcWRKY26 cascade is a missing link in the jasmonate-activated expression of taxol biosynthesis gene DBAT in *Taxus chinensis*. Int. J. Mol. Sci..

[40] Cao X., Xu L., Li L. (2022). TcMYB29a, an ABA-responsive R2R3-MYB transcriptional factor, upregulates taxol biosynthesis in *Taxus chinensis*. Front. Plant Sci..

[41] Ren Y., Liu D., Zhao W., Wang X., Cao X., Wan W. (2025). TcMYB73, a salicylic acid-responsive R2R3-MYB transcription factor, positively regulates paclitaxel biosynthesis in *Taxus chinensis* in direct and indirect ways. BMC Plant Biol..

[42] Yu C., Huang J., Wu Q. (2022). Role of female-predominant MYB39-bHLH13 complex in sexually dimorphic accumulation of taxol in *Taxus media*. Hortic. Res..

[43] Sun M.-S., Jia Y., Chen X.Y., Chen J.S., Guo Y., Fu F.F., Xue L.J. (2024). Regulatory microRNAs and phasiRNAs of paclitaxel biosynthesis in *Taxus chinensis*. Front. Plant Sci..

[44] Huang Q., Roessner C.A., Croteau R., Scott A.I. (2001). Engineering *Escherichia coli* for the synthesis of taxadiene, a key intermediate in the biosynthesis of taxol. Bioorg. Med. Chem..

[45] Martin V.J.J., Pitera D.J., Withers S.T., Newman J.D., Keasling J.D. (2003). Engineering a mevalonate pathway in *Escherichia coli* for production of terpenoids. Nat. Biotechnol..

[46] Ajikumar P.K., Xiao W.H., Tyo K.E.J., Wang Y., Simeon F., Leonard E., Mucha O., Phon T.H., Pfeifer B., Stephanopoulos G. (2010). Isoprenoid pathway optimization for Taxol precursor overproduction in *Escherichia coli*. Science.

[47] Bian G., Yuan Y., Tao H., Shi X., Zhong X., Han Y., Fu S., Fang C., Deng Z., Liu T. (2017). Production of taxadiene by engineering of mevalonate pathway in *Escherichia coli* and endophytic fungus *Alternaria alternata* TPF6. Biotechnol. J..

[48] Wang J.Y., Huang Z.Y., Wu Q.Y., Pan J., Li C.X., Xu J.H. (2022). Facile biosynthesis of taxadiene by a newly constructed *Escherichia coli* strain fusing enzymes taxadiene synthase and geranylgeranyl pyrophosphate synthase. Process Biochem..

[49] Engels B., Dahm P., Jennewein S. (2008). Metabolic engineering of taxadiene biosynthesis in yeast as a first step towards Taxol (paclitaxel) production. Metab. Eng..

[50] Nowrouzi B., Li R.A., Walls L.E., d’Espaux L., Malci K., Liang L., Jonguitud-Borrego N., Lerma-Escalera A.I., Morones-Ramirez J.R., Keasling J.D. (2020). Enhanced production of taxadiene in *Saccharomyces cerevisiae*. Microb. Cell Fact..

[51] Walls L.E., Malci K., Nowrouzi B., Li R.A., d’Espaux L., Wong J., Dennis J.A., Semião A.J.C., Wallace S., Martinez J.L. (2021). Optimizing the biosynthesis of oxygenated and acetylated Taxol precursors in *Saccharomyces cerevisiae* using advanced bioprocessing strategies. Biotechnol. Bioeng..

[52] Malci K., Santibanes R., Jonguitud-Borrego N., Santoyo-Garcia J.H., Kerkhoven E.J., Rios-Solis L. (2023). Improved production of Taxol precursors in *Saccharomyces cerevisiae* using combinatorial in silico design and metabolic engineering. Microb. Cell Fact..

[53] Zhang Z., Huang L., Zhang Z.J., Xu J.H., Yu H.L. (2023). Rational design of taxadiene hydroxylase by ancestral enzyme construction and the elucidation of key amino acids. Biochemistry.

[54] Abdallah I.I., Pramastya H., van Merkerk R., Sukrasno, Quax W.J. (2019). Metabolic engineering of *Bacillus subtilis* toward taxadiene biosynthesis as the first committed step for taxol production. Front. Microbiol..

[55] Xu M., Xie W., Luo Z., Li C.X., Hua Q., Xu J.H. (2023). Improving solubility and copy number of taxadiene synthase to enhance the titer of taxadiene in *Yarrowia lipolytica*. Synth. Syst. Biotechnol..

[56] Zhong J., Wang Y., Chen Z., Yalikun Y., He L., Liu T., Ma G. (2024). Engineering cyanobacteria as a new platform for producing taxol precursors directly from carbon dioxide. Biotechnol. Biofuels Bioprod..

[57] Besumbes O., Sauret-Güeto S., Phillips M.A., Imperial S., Rodríguez-Concepción M., Boronat A. (2004). Metabolic engineering of isoprenoid biosynthesis in *Arabidopsis* for the production of taxadiene, the first committed precursor of Taxol. Biotechnol. Bioeng..

[58] Fu J., Xu W., Huang W., Wang B., Li S., Zhang J., Chang L. (2021). Importation of taxadiene synthase into chloroplast improves taxadiene production in tobacco. Planta.

[59] Perez-Matas E., Hidalgo-Martinez D., Moyano E., Palazon J., Bonfill M. (2024). Overexpression of BAPT and DBTNBT genes in *Taxus baccata* in vitro cultures to enhance the biotechnological production of paclitaxel. Plant Biotechnol. J..

[60] DeJong J.M., Liu Y., Bollon A.P., Long R.M., Jennewein S., Williams D., Croteau R.B. (2006). Genetic engineering of taxol biosynthetic genes in *Saccharomyces cerevisiae*. Biotechnol. Bioeng..

[61] Karaca H., Kaya M., Kapkac H.A., Levent S., Ozkay Y., Ozan S.D., Nielsen J., Krivoruchko A. (2024). Metabolic engineering of *Saccharomyces cerevisiae* for enhanced taxadiene production. Microb. Cell Fact..

[62] Zhang C., Wang J., Shi Y., Wu N., Li X., Wang Y., Li B., Xiao W., Yao M., Yuan Y. (2024). Improving the expression of taxadiene synthase to enhance the titer of taxadiene in *Saccharomyces cerevisiae*. Green Chem..

[63] Ma Y., Shang Y., Stephanopoulos G. (2024). Engineering peroxisomal biosynthetic pathways for maximization of triterpene production in *Yarrowia lipolytica*. Proc. Natl. Acad. Sci. USA.

[64] Wlodarczyk A., Gnanasekaran T., Nielsen A.Z., Zulu N.N., Mellor S.B., Luckner M., Thøfner J.F.B., Olsen C.E., Burow M. (2016). Metabolic engineering of light-driven cytochrome P450-dependent pathways into *Synechocystis* sp. PCC 6803. Metab. Eng..

[65] Anterola A., Shanle E., Perroud P.F., Quatrano R. (2009). Production of taxa-4(5),11(12)-diene by transgenic *Physcomitrella patens*. Transgenic Res..

[66] Escrich A., Hidalgo D., Bonfill M. (2023). Polyploidy as a strategy to increase taxane production in yew cell cultures: Obtaining and characterizing a *Taxus baccata* tetraploid cell line. Plant Sci..

[67] Park Y.K., Nicaud J.M., Ledesma-Amaro R. (2023). What makes *Yarrowia lipolytica* well suited for industry?. Trends Biotechnol..

